# Hydrogen Bond Donors Dictate the Frictional Response in Deep Eutectic Solvents

**DOI:** 10.1021/acs.langmuir.3c03303

**Published:** 2024-03-06

**Authors:** Hannah J. Hayler, James E. Hallett, Susan Perkin

**Affiliations:** † Physical and Theoretical Chemistry Laboratory, Department of Chemistry, University of Oxford, Oxford OX1 3QZ, U.K.; ‡ Department of Chemistry, School of Chemistry, Food and Pharmacy, University of Reading, Whiteknights Campus, Reading RG6 6AD, U.K.

## Abstract

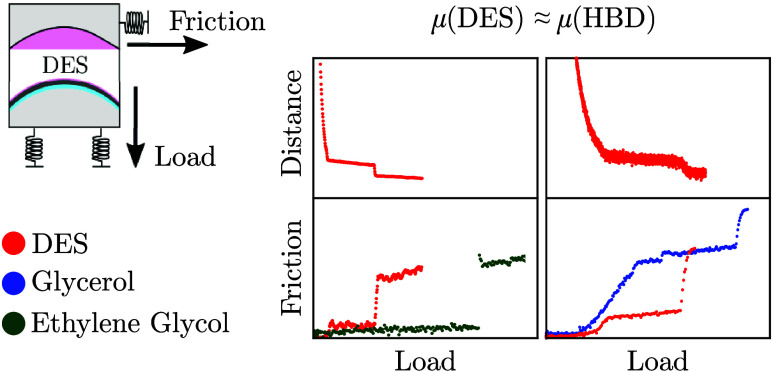

Deep eutectic solvents (DESs) show promise as boundary lubricants between sliding surfaces, taking advantage of their physical stability, chemical stability, and tunability. Here, we study friction forces across nanofilms of two archetypal DES mixtures: choline chloride + ethylene glycol and choline chloride + glycerol. Using a surface force balance, we control the film thickness (to subnanometer precision) and determine the friction force simultaneously. Measurements are made at different mole fractions of the choline chloride salt and the molecular solvent, allowing us to determine the role of each species in the observed behavior. We find that the nature of the molecular solvent is dominant in determining the lubrication behavior, while the fraction of ChCl is relatively less important. By analyzing the steps in friction and the gradient of friction with load as the layers squeeze away from between the surfaces, we learn various mechanistic aspects of lubrication across the DES nanofilms of relevance to design and optimization of these promising fluids.

## Introduction

The term deep eutectic solvent (DES) has been used to describe mixtures, typically involving a salt and a complexing molecular solvent, with substantial depression of the eutectic temperature compared to ideal behavior.^1^ The heuristic understanding that the molecular species in a DES complex with the ions led to their frequent consideration as a subclass of ionic liquids. In an ionic liquid (IL), the presence of large, asymmetric ions frustrate crystallization and lead to salt melting temperatures around room temperature.^2^ DESs also contain ion pairs, but the presence of a molecular solvent results in a 2-component system.^3^ Like ILs, the size and shape of the ions can influence the phase behavior, and also, the presence of a hydrogen bond-capable molecular solvent can lead to strong hydrogen bonding between the components, which favor the liquid phase at the eutectic composition, leading to their name.^4^ DESs are commonly formed from the complexation of a quaternary ammonium salt like choline chloride and hydrogen bond donors (HBDs), e.g., ethylene glycol, glycerol, and urea.^5^

Both DESs and ILs have desirable solvent properties, including tunability, wide liquidus ranges, and high thermal stability.^5^ DESs can also be advantageous over ILs as the components can be biodegradable, nontoxic, and inexpensive.^6^ In fact, these advantages have led to DESs being termed “greener” solvent alternatives^7,8^ with uses in synthesis and metal processing.^5,9^ DESs have also been suggested as promising lubricants.^10−13^ Of particular interest is within the marine industry, where it is preferable to have water-miscible and biodegradable lubricants that do not have adverse effects on marine water systems or contribute to water pollution.^11^ The lubricative properties of DESs have been studied previously, with a focus on bulk measurements across different surfaces.^10−13^ It has also been suggested that lubrication in DESs may be enhanced at the eutectic composition.^11,12^ Recently, friction forces across 1:2 choline chloride and ethylene glycol nanofilms were studied, and the influence of water content was quantified.^14^

In this work, we used a surface force balance (SFB) to study the lubricative properties of two archetypal DESs: (i) choline chloride + ethylene glycol and (ii) choline chloride + glycerol, under nanoconfinement. As control experiments for comparison, we also studied the pure molecular HBDs, i.e., ethylene glycol and glycerol. We explore how the frictional response changes with the choline chloride concentration to understand the role of the ions. We also compare the frictional behavior of the DESs with the pure HBDs to determine the contribution from the HBD to the lubricative properties of the DES.

## Experimental Section

**Figure 1:**
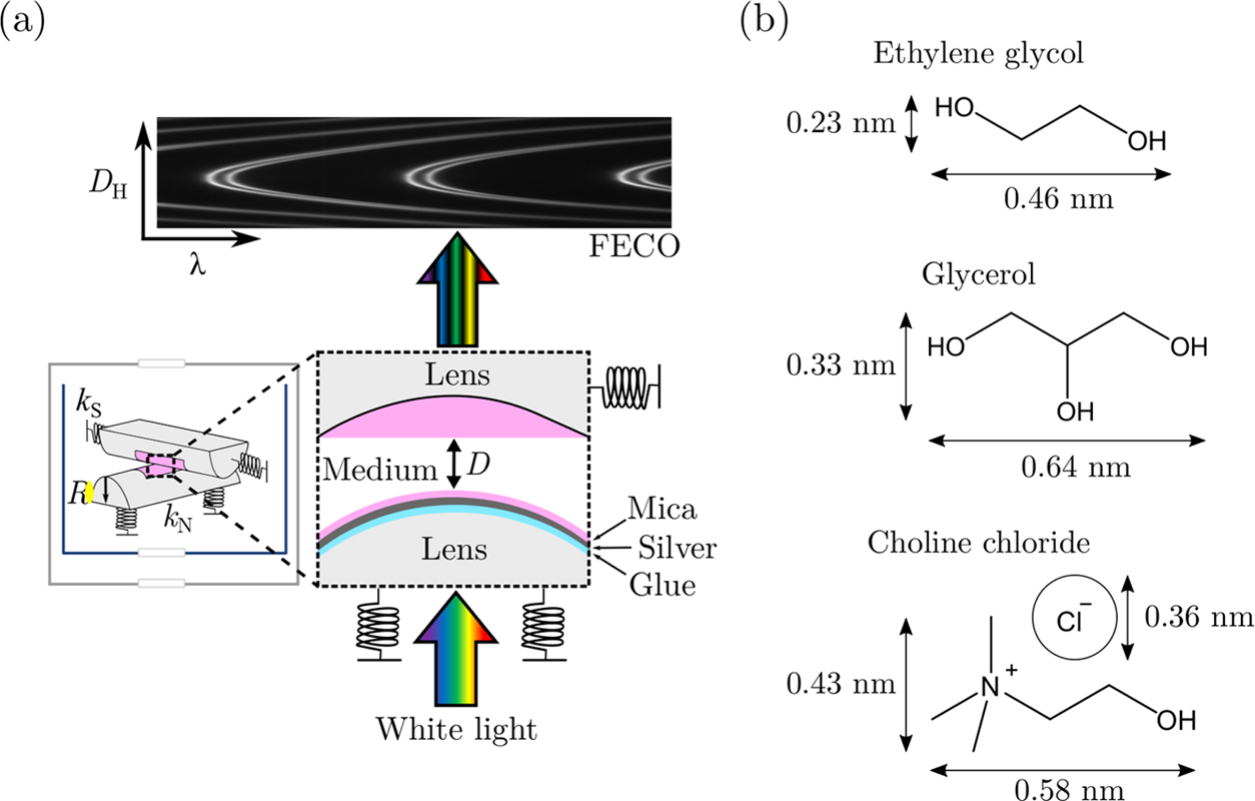
(a) Schematic of the surface force balance. White light is passed through the bottom lens into the interferometric cavity. Wavelengths that constructively interfere pass through the top lens to the spectrometer and then the camera, yielding fringes of equal chromatic order (FECO). The FECO are used to determine surface separation, *D*, and the radius of curvature of the lenses, *R*, using the position of the fringes in wavelengths, λ, and the in-plane (horizontal) distance, *D*_H_. Normal and shear springs with spring constants, *k*_N_ and *k*_S_, are used to determine the normal and frictional forces, respectively. (b) Molecular structure and approximate dimensions of ethylene glycol, glycerol, and choline chloride as obtained from measurements across van der Waals structures simulated in Gaussian.^3,18^

Experiments were performed using an SFB, as shown in Figure 1(a). The liquid is confined between two surfaces that are mounted in a crossed-cylindrical configuration. In this work, both surfaces are made of atomically smooth muscovite mica (typically 2–4 μm) of equal thicknesses that have been back-silvered and glued (silver-side down) onto a hemicylindrical lens (radius of curvature ≈1 cm). The silver-mica-medium-mica-silver stack forms an interferometric cavity. By shining white light through the bottom lens and into the interferometric cavity, only wavelengths that interfere constructively between the two silver surfaces will pass through the cavity and out to a spectrometer, yielding fringes of equal chromatic order (FECO). The thickness of the optical cavity (mica-medium-mica) can be determined by first calibrating the mica thickness (in air) and then by measuring the shift of the FECO as the lenses are moved closer or further away from one another to yield the distance, *D*. Normal and lateral motion is enabled by mounting one lens on a piezoelectric tube. Both lenses are mounted on a set of leaf springs of known spring constants, the deflection of which can be used to determine the normal, *F*_N_, and shear forces, *F*_S_. Further information regarding the SFB technique is detailed elsewhere.^15−17^

We study DESs and molar ratios as follows (structures shown in Figure 1(b)): Choline chloride (ChCl) + ethylene glycol (EG) at ChCl/EG molar ratios of 1:2, 1:3, and 1:10 and ChCl + glycerol (Gly) at a ChCl/Gly molar ratio of 1:2. Note that the DES mixtures at a molar ratio of 1:2 in each case have in the past been considered to be the eutectic compositions;^10,11^ however, it has also been suggested that the eutectic points are not at this precise point.^19,20^ Ethylene glycol (Acros, anhydrous, 99.8%), glycerol (Sigma-Aldrich, anhydrous, ≥99.5%), and choline chloride (Sigma-Aldrich, anhydrous, ≥99%) were used as received. 1:2 mixtures were prepared by mixing 1 mol equivalent of ChCl with 2 mol equivalents of EG or Gly. The resulting mixtures were stirred overnight at 60 °C under a nitrogen atmosphere and the homogeneous liquid formed was subsequently stored under nitrogen. Off-eutectic 1:3 and 1:10 ChCl/EG samples were prepared and stirred overnight at 60 °C at room temperature and pressure.

## Results and Discussion

Normal forces across thin films of EG,^3,21^ Gly,^3^ 1:2 ChCl/EG,^3,14,22^ and 1:2 ChCl/Gly^3,22^ have been measured previously, and it is understood that the observed oscillatory, or “structural”, forces originate from molecular layers being “squeezed out” from the confined region. Using dimensional arguments, the squeeze-out thicknesses can be attributed to the confined structure. We started by checking that the observed normal forces corroborate earlier studies,^3,14,21,22^ which was indeed the case: normal force profiles can be found in the Supporting Information. After this control step, we focused on studying the shear forces across the fluids.

**Figure 2:**
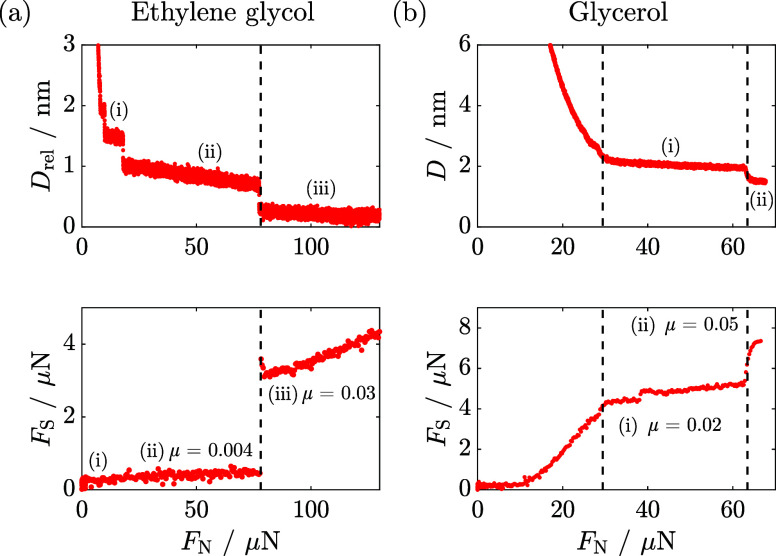
Representative surface separation, *D* (top plot), and shear force, *F*_S_ (bottom plot), as a function of increasing normal force, *F*_N_, across dry (a) ethylene glycol and (b) glycerol. The vertical dashed lines highlight the simultaneous discrete steps in surface separation and shear force when the normal force exceeds certain critical values and corresponds to discontinuous squeezing-out of molecular layers from between the surfaces as they are pressed together. (a) Top plot*:* Surface separation is given relative to the position of the closest approach, *D*_rel_. Bottom plot*:* No friction coefficient can be extracted for layer (i). The friction coefficient of layer (ii) is μ = 0.004. The friction coefficient of layer (iii) is μ = 0.03. (b) Top plot: Surface separation is given relative to calibration of *D* = 0 at the mica–mica contact. Bottom plot: The friction coefficient of layer (i) is μ = 0.02. The friction coefficient of layer (ii) is μ = 0.05. The frictional responses shown are representative of multiple repeat measurements and are qualitatively reproducible. It should be noted that small changes in composition, mica alignment, temperature, and contact geometry can influence the calculated friction coefficients by up to a factor of 2.^14^

We begin by discussing the lubricative properties of the pure molecular liquids ethylene glycol (EG) and glycerol (Gly). Friction coefficients across ethylene glycol^23^ and glycerol^24−29^ have been measured in the past, but here we study these liquids under nanoconfinement with subnanometer precision in film thickness; this allows for investigation of the relation between the number of molecular layers in the film and the frictional characteristics and will allow for direct comparison with the DES mixtures in due course. The frictional response of dry ethylene glycol and glycerol is shown in Figure 2(a,b), respectively. The surface separation, *D* (top), and shear force, *F*_S_ (bottom), as a function of increasing normal force, *F*_N_, are shown for both systems. At large separations (low *F*_N_), there is a negligible shear force, i.e., only the imposed lateral motion is observed with no coupling between the confining surfaces. As the surface separation decreases (red curves), we observe a series of discontinuities in the *F*_S_–*F*_N_ profiles and a simultaneous increase in the amplitude of *F*_S_. These steps in *D* and *F*_S_ as the film is compressed are attributed to sudden squeeze-out of individual molecular layers. By measurement of the surface separation and shear forces concurrently, any changes in the observed frictional response can be directly attributed to the change in film thickness and structure.

Within each layer, i.e., between the steps or discontinuities, *F*_S_ increases linearly with *F*_N_. The gradient of each of these linear regions is interpreted as a friction coefficient, μ, particular to the film thickness. For ethylene glycol in Figure 2(a), we observe an increase from μ = 0.004 to μ = 0.03 as molecular layers are squeezed out from layer (ii) to (iii). On the other hand, for glycerol, we observe friction coefficients that are comparable between different layers, and greater confinement only influences the adhesive contribution to *F*_S_ (see Figure 2(b)). That is to say, the magnitude of *F*_S_ increases with *F*_N_ but  remains approximately constant except during the layer transitions. For glycerol, in layer (i), a small step is apparent in the *F*_S_–*F*_N_ profile at ≈40 μN without a corresponding change in the layer index in the top plot. This is likely due to a molecular rearrangement within the layer as the load increases, resulting in a more ordered structure with a greater adhesive force between the surfaces. A further point of note in comparing the behavior of EG and Gly is that the layering in EG is more prominent than the layering in Gly. First, Gly is more viscous than EG, so we expect a significant viscous force to contribute to the overall force observed. Even at the slowest approach rates possible in our experiments, the viscosity contributes to the inability to push through to deeper layers (below approximately 1.75 nm) and much larger normal forces are required to access the innermost layer (see the SI). Second, EG has a more linear structure than Gly that lends itself well to layering (into a more “2D-like” structure). The extra hydroxyl group on Gly is likely to create a more “3D-like” hydrogen-bonding network; it is likely that Gly has greater interlayer interactions, which leads to a “stiffening” of the layers in response to shear, increasing interlayer friction. Indeed, the hydrogen-bonding networks in the two fluids are likely very important in determining their structure and properties in the thin films. Gly has three hydroxyl groups per molecule while ethylene glycol has two, each of which can act as a double proton donor and a double proton acceptor, forming up to six and four hydrogen bonds, respectively, leading to significant hydrogen-bonding network formation in the pure liquids. However, in an interfacial geometry, the molecules are orientationally constrained, which can lead to a different network structure. Nevertheless, hydrogen bonding is still expected to be strong and the additional hydrogen-bonding propensity of glycerol may increase the interlayer interactions cf. EG.

**Figure 3:**
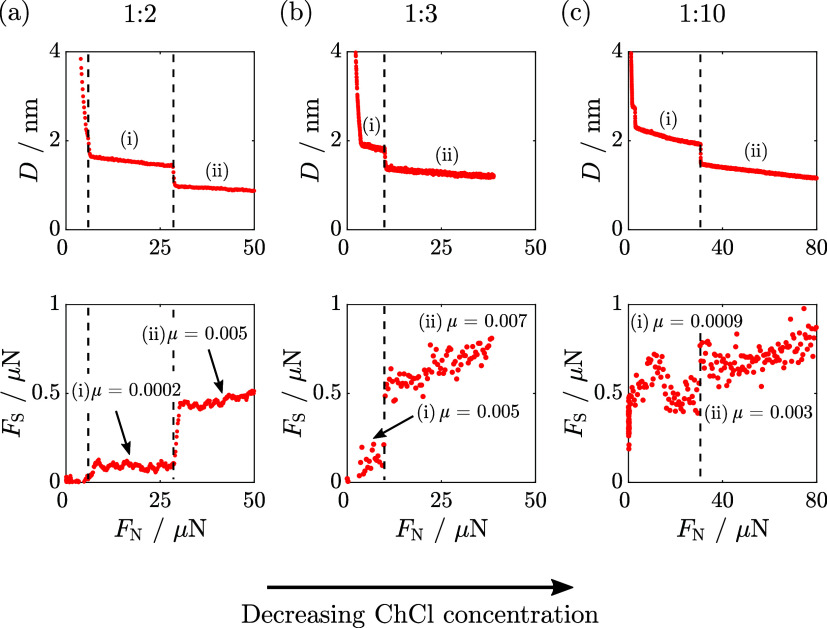
Representative surface separation, *D* (top plot), and shear force, *F*_S_ (bottom plot), as a function of increasing normal force, *F*_N_, across (a) 1:2,^14^ (b) 1:3, and (c) 1:10 choline chloride/ethylene glycol (ChCl/EG). The vertical dashed lines highlight the simultaneous discrete steps in surface separation and shear force with normal force. (a) Bottom plot: The friction coefficient of layer (i) is μ = 0.0002. The friction coefficient of layer (ii) is μ = 0.005. (b) Bottom plot: The friction coefficient of layer (i) is μ = 0.005. The friction coefficient of layer (ii) is μ = 0.007. (c) Bottom plot: The friction coefficient of layer (i) is μ = 0.0009. The friction coefficient of layer (ii) is μ = 0.003. The frictional responses shown are representative of multiple repeat measurements and are qualitatively reproducible. It should be noted that small changes in composition, mica alignment, temperature, and contact geometry can influence the calculated friction coefficients by up to a factor of 2.^14^ Furthermore, we note that the scatter within a single run (random error arises from ambient building vibrations and noise in the environment). The distinctly lower noise in (a) compared to those in (b, c) arose because these measurements were made during a period of the COVID-19 pandemic when the building and surroundings presented particularly low noise.

We next consider the role of the cholinium and chloride ions on the lubricative properties of DES mixtures by comparison to the pure molecular liquids. We study near-eutectic mixtures for both EG- and Gly-based mixtures and focus on the EG-based mixtures for off-eutectic compositions due to their lower viscosity, which facilitates observation of the layer details. Figure 3 shows the frictional response across three compositions of ChCl/EG: (a) 1:2, (b) 1:3, and (c) 1:10. The behavior of (1:2) ChCl/EG has been reported previously and is reproduced here to aid in comparison.^14^ Surprisingly, under the loads studied, we find that the friction coefficients across 1:2, 1:3, and 1:10 ChCl/EG are comparable to one another, and we do not see a significant dependence on the choline chloride concentration. (Note that, due to variations in mica twist angle and small changes in temperature and geometry, the friction coefficients in repeat experiments can vary by a factor of ca. 2 between experiments. However, within a single experiment, the friction measured across different layers is substantially more precise than this, with ca. 10% error.) In each case, Figure 3(a–c), the steps indicating layer transitions arise at similar values of applied load and the magnitude of friction force is similar.

**Figure 4:**
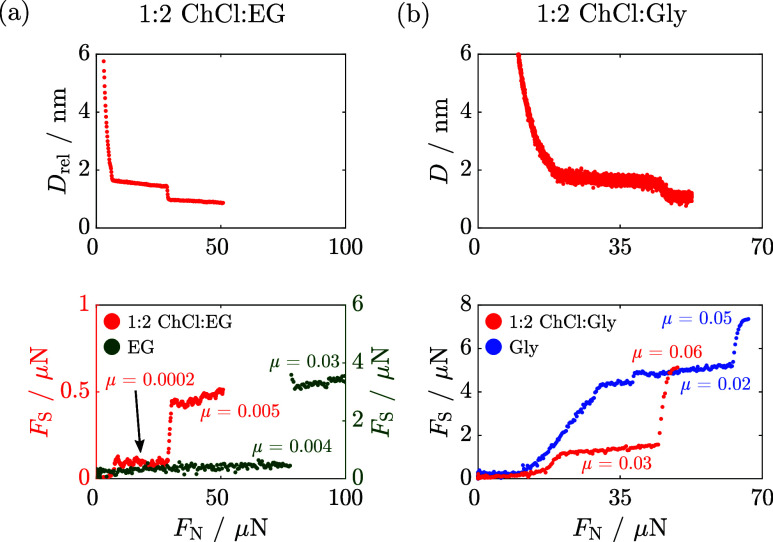
Representative surface separation, *D* (top plot), and shear force, *F*_S_ (bottom plot), as a function of increasing normal force, *F*_N_, across dry 1:2 (a) choline chloride/ethylene glycol (ChCl/EG)^14^ and (b) choline chloride/glycerol (ChCl/Gly). (a) Top plot: Surface separation is given relative to the position of the closest approach, *D*_rel_. Bottom plot: *F*_S_–*F*_N_ profiles across 1:2 ChCl/EG (red, left axis) and pure EG (green, right axis). At *F*_N_ < 80 μN, the friction coefficients across 1:2 ChCl/EG and pure EG are comparable. (b) Bottom plot: *F*_S_–*F*_N_ profiles across 1:2 ChCl/Gly (red) and pure Gly (blue). The friction coefficients across 1:2 ChCl/Gly and pure Gly are comparable under the loads reported here. The frictional responses shown are representative of multiple repeat measurements and are qualitatively reproducible. It should be noted that small changes in composition, mica alignment, temperature, and contact geometry can influence the calculated friction coefficients by up to a factor of 2.^14^

Finally, in order to check the role of the molecular component on the lubricative behavior of DESs, we investigate the frictional response across two choline chloride-based DESs with different molecular components: results for (a) 1:2 ChCl/EG and (b) 1:2 ChCl/Gly are compared in Figure 4. The responses of pure EG and Gly (from Figure 2) are also plotted for comparison. In both EG and 1:2 ChCl/EG, the coefficient of friction, μ, increases by an order of magnitude as the liquid layers squeeze out. In contrast to this, in pure Gly and in the 1:2 ChCl/Gly, the friction coefficients remain the same (within a factor of 2),^14^ regardless of load or film thickness. Interestingly, there is a strong similarity between the behavior of pure molecular components and their corresponding DESs, yet there is a clear distinction between the EG-based and Gly-based systems. That is to say, the molecular component (EG, Gly) is the most significant in determining the lubricative properties of these DESs, and there seems to be a distinct difference in the mechanism between the two.

In order to discuss these mechanisms, we recall that boundary friction across liquid films can be captured by the following approximate expression
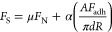
1where μ is the load-controlled friction coefficient, α is the adhesion-controlled friction coefficient, *A* is the flat contact area, *F*_adh_ is the adhesion force between the surfaces, and *d* is the distance between shearing liquid layers.^30,31^ The above expression may apply to average properties for nonuniform films. In the particular case of measurements across nanofilms with sufficient resolution to observe the behavior of distinct molecular layers, the expression can be modified with an index *i* to indicate that each parameter in eq 1 may depend on the precise number of molecular layers, *i*
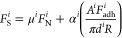
2The discontinuities in *F*_S_–*F*_N_ observed in all our measurements presented here are an inherent property of liquids that exhibit oscillatory normal forces (see *D*–*F*_N_ profiles, including in the SI), on account for the adhesive force between the surfaces changing with the number of molecular layers.^32^ For this reason, we can interpret the step changes in *F*_S_ when the film thickness changes suddenly as largely due to a step change in adhesive force (and thus the second, adhesion, term in eq 2) when an additional layer is squeezed out of the film. On the other hand, the slope of *F*_S_–*F*_N_ in between steps is interpreted with a load-controlled friction coefficient. With this in mind, we note the following interpretations from our measurements presented here: (i) In both EG and its mixtures with ChCl, the load-controlled friction coefficient increases as the film thickness decreases. This increase in μ can be attributed to an increased activation barrier to sliding across the film, likely due to greater inter- and intralayer ordering nearer the mica surface and/or a change in the active shear plane.^14,31^ (ii) Over a wide range of concentrations, the ChCl/EG mixtures exhibit similar load-controlled friction, implying that the molecular ordering and alignment on the mica surface, and its dependence on film thickness, are largely determined by the EG properties and not the ChCl. This may imply that ChCl is partly or largely excluded from the first layer adjacent to the mica surface. Alternatively, it may be that the interfacial concentration of ChCl remains similar across the wide range of concentrations studied despite varying bulk concentrations. (iii) In both Gly and its mixture with ChCl, the load-controlled friction coefficient is remarkably unchanged when layers of fluid are squeezed out from the film, implying a similar sliding mechanism, regardless of load or film thickness. This may be due to the weakest sliding plane laying at or near the mica–liquid interface, rather than at the midplane between the surfaces since this is the region less altered when a layer transition takes place. (iv) In all cases of the pure EG and Gly and DESs studied, the load-controlled friction coefficients are rather low, indicating very good lubricity, to such an extent that the adhesion-controlled contribution to friction (second term in the equations above) is the dominant term. Thus, tuning interfacial adhesion (wetting) of the lubricant may be an important avenue when optimizing lubrication by DES films.

## Conclusions

In summary, we have studied the lubricative properties of EG, Gly, and their corresponding choline-chloride-based DESs using the SFB. All fluids studied demonstrated low coefficients of friction and low absolute friction forces. We find that under nanoconfinement, the concentration of ChCl ions (over the range studied) does not seem to influence the frictional response of the mixture. We also show that the frictional behavior of the DESs studied is largely dominated by the properties of the molecular component and is different in character for EG and Gly lubricants. However, the presence of ChCl allows for use in applications that require an electrolytic lubricant, e.g., electrotunable friction,^33^ or other application-specific tuning of properties, e.g., vapor pressure or surface interactions. Overall, we demonstrate that the choice of molecular component is important for lubrication with DESs, and our molecular resolution studies allowed us to unpick several aspects of the lubrication mechanism, which should contribute to designing DES-based lubricants in the future.

## Supporting Information Available

The following files are available free of charge. The Supporting Information is available free of charge at https://pubs.acs.org/doi/10.1021/acs.langmuir.3c03303.

SI: Normal force profiles across ethylene glycol, glycol, 1:2 choline chloride/glycerol, 1:2 choline chloride/ethylene glycol, 1:3 choline chloride/ethylene glycol, and 1:10 choline chloride/ethylene glycol (PDF)
