# Supplementary material for: Hydrogen Bond Donors Dictate the Frictional Response in Deep Eutectic Solvents
Source: Langmuir. 2024 Mar 6;40(11):5695–700. doi: 10.1021/acs.langmuir.3c03303 (PMC10956492; doi:10.1021/acs.langmuir.3c03303)
Supplement: Supplementary file 1 — la3c03303_si_001.pdf [file la3c03303_si_001.pdf]

Supporting Information

Hydrogen bond donors dictate the frictional response in deep eutectic solvents

Hannah J. Hayler,† James E. Hallett,‡ and Susan Perkin*,†

†*Physical and Theoretical Chemistry Laboratory, Department of Chemistry, University of Oxford, Oxford, OX1 3QZ, UK* ‡*Department of Chemistry, University of Reading, Reading, UK*

E-mail: susan.perkin@chem.ox.ac.uk

Normal Forces: Hydrogen Bond Donors

Ethylene glycol

A representative normal force profile across dry ethylene glycol (EG) is shown in Figure S1(a). Within 5 nm of the closest approach, an oscillatory force is observed. In Figure S1(a), the series of repulsive hard walls followed by a 'jump-in' point to smaller surface separation (due to the spring instability when $\partial F_{\mathrm{N}}(D)/\partial D \geq k_{\mathrm{N}}$) are indicative of a layered structure between the surfaces. The separation between the hard walls in dry ethylene glycol is 0.38 $\pm$ 0.09 nm and corresponds to the long dimension of an ethylene glycol molecule, corroborating with literature measurements.[1,2]

The inset of Figure S1(a) shows the long-range normal force decaying exponentially with surface separation according to $F_{\mathrm{N}}/R \propto \exp(-D_{\mathrm{rel}}/\lambda_{\mathrm{exp}})$, where λ_{exp} is the experimentally observed decay length. In dry ethylene glycol, $\lambda_{\mathrm{exp}} = 7.9 \pm 0.3$ nm. From Debye-Hückel theory, the observed decay length corresponds to a monovalent ion concentration of 0.8 mM. Ions present in the confined region are likely due to the dissolution of potassium ions from the mica surface,[3] impurities in the EG sample, or other dissolved ions from the environment or cleaning procedures. It could be suggested that excess protons from the dissociation of ethylene glycol contribute to the ionic strength in the confined region, however at room temperature the $\mathrm{pK_a} = 15.1$[4] so we expect minimal dissociation.

Glycerol

An oscillatory force within 5 nm of zero surface separation is observed in Figure S1(b) indicating a layered structure between the surfaces. The separation between the hard walls in dry glycerol (Gly) is 0.44 ± 0.01 nm. It should be noted that within the layers there is a measurable compression *i.e.* we remain in a layer with increasing load where D decreases continuously and no discrete jump-in point is observed. The compression is observed as a finite gradient between the jumps. For example, in the inset of Figure S1(b), the layer at $\approx$ 3 nm compresses by $\Delta_{\mathrm{C}} \approx 0.2$ nm, even under low normal load. Compression can be attributed to deformation of the mica surfaces and/or squeezing the molecular layers.[5] As the measurements in Figure S1(b) were completed with thin mica (< 3 μm), the compression observed here can be attributed to the latter. Considering both the jump-in separation, Δ_{J}, and compression yields a dimension, $\Delta_{\mathrm{J}} + \Delta_{\mathrm{C}} \approx$ 0.65 nm, which corresponds with the long dimension of a glycerol molecule. The final jump-in point to $D = 0$ nm was measurably thinner, 0.27 ± 0.02 nm, and is likely due to the glycerol molecules lying flat against the mica surface. These results corroborate the work of Chen *et al.*[2]

Normal Forces: Deep Eutectic Solvents

1:2 choline chloride:glycerol

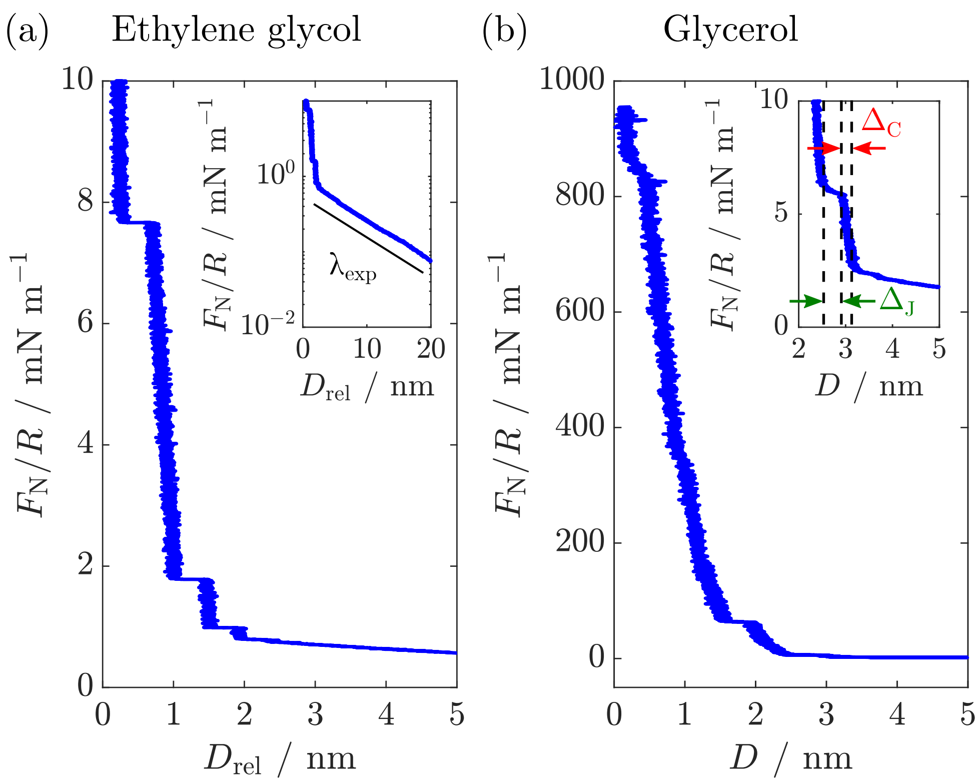

Figure S1 Representative normal force, F_N, normalised by the radius of curvature, R, as a function of surface separation, D, profiles in (a) ethylene glycol and (b) glycerol. (a) Surface separation is given relative to the position of closest approach, D_{rel}. The inset shows a log-linear representation of the normal force on approach. The solid black line indicates the gradient of the long-range exponential decay where $\lambda_{exp} = 7.9 \pm 0.3$ nm. (b) The inset shows the structural forces when $F_N/R \leq 10$ mN m^{-1}. The jump-in separation, Δ_J, and layer compression, Δ_C, are shown in green and red, respectively. Higher loads are accessed by using a stiffer normal spring.

The force profile in Figure S2(a) shows oscillatory forces indicative of a layering structure between the surfaces in dry 1:2 choline chloride (ChCl):Gly samples. The force profile shows two steps, one with a weaker amplitude at $F_N/R < 1$ mN m^{-1} and one with a larger amplitude at $F_N/R \approx 5-7$ mN m^{-1}. The two steps are separated by a region of continuous squeeze-out. The step dimension is $\Delta_J = 0.33 \pm 0.02$ nm, whilst the squeeze-out extends over $\Delta_C \approx 1$ nm. In dry 1:2 ChCl:Gly, the layer thickness corresponds well with the short dimension of the glycerol molecule.

Neutron diffraction measurements and simulations of 1:2 ChCl:Gly by Turner and Holbrey [6] indicated the network of Gly-Gly hydrogen bonds present in pure Gly is retained in 1:2 ChCl:Gly. We therefore suggest that the steps (Δ_J) observed in the dry sample are predominantly due to glycerol molecules.

The point of closest approach was at $D = 1$ nm. We propose that the point of closest approach observed corresponds to the thickness of an immovable solid-like layer of molecules at the surface that cannot be squeezed out over the normal loads applied here. In pure glycerol, the final observed layer (before reaching mica-mica contact) also sat at $D \approx 1$ nm, and was only removed at higher loads, $F_N/R \approx 800$ mN m^{-1}.

1:2 choline chloride:ethylene glycol

The normal force profile across dry 1:2 ChCl:EG is shown in Figure S2(b) and is reproduced from Hallett *et al.*[7] We observe an oscillatory force at $D < 3$ nm, indicative of molecular layering between the surfaces. The separation between the hard walls is uniform with a dimension of 0.45 $\pm$ 0.03 nm. Using dimensional

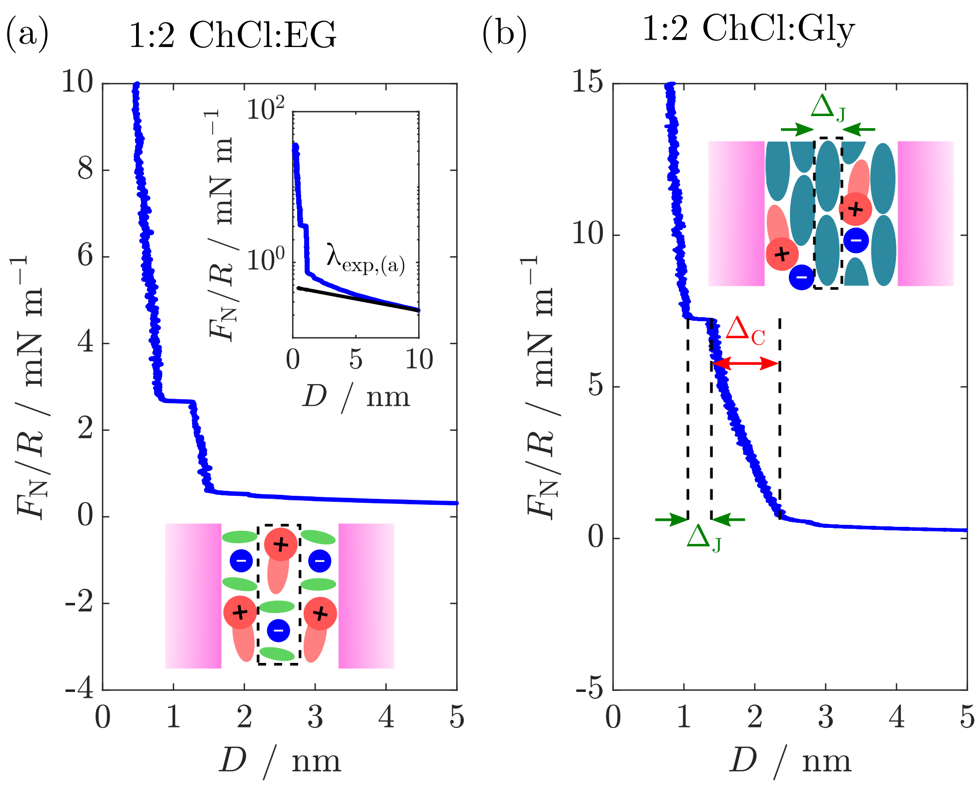

Figure S2 Representative normal force, F_N, normalised by the radius of curvature, R, as a function of surface separation, D, profiles in dry (a) 1:2 ChCl:Gly and (b) 1:2 ChCl:EG. Proposed confined structure schematics determined using dimensional arguments are shown. The mica surfaces are shown in pink, glycerol molecules in blue-grey, choline cations in red, chloride anions in blue, and ethylene glycol molecules in green. The dashed black box indicates the squeeze-out repeat unit giving rise to steps in the force-distance profile. (a) The jump-in separation, Δ_J, and layer compression, Δ_C, are shown in green and red, respectively. (b) Reproduced from Hallett *et al.*[7] The inset shows a log-linear representation of the normal force on approach. The solid black line indicates the gradient of the long-range exponential decay where $\lambda_{exp,(b)} = 12 \pm 1$ nm.

arguments, the steps can be attributed to the larger dimension of the choline ions lying parallel to the mica surface. In ionic liquids the wavelength of the oscillation typically matches the ion-pair dimension, implying that electroneutral pairs of layers are expelled.[8] Whereas, in the present experiments, electroneutral layers do not correspond to the ion-pair dimension and are instead consistent with a single layer containing all three constituents: choline cations, chloride anions, and EG. This 'checkerboard-like' behaviour has also been reported in ionic liquids,[9–11] and allows electroneutrality to be maintained without templating into separate cation and anion-dominated layers. This corroborates molecular dynamics simulations by Mamme *et al.*,[12] where they observe layers of mixed composition beyond the Stern layer.

The positions of the layers on successive measurements and separate experiments are highly reproducible, but the magnitude of F_N/R can vary due to small differences in DES composition and the charge on the mica.

The inset of Figure S2(b) shows the long-range normal force decaying exponentially with surface separation with an experimental decay length, $\lambda_{exp,(a)} = 12 \pm 1$ nm. From Debye-Hückel theory (using $\epsilon_r = 32$),[13] the predicted Debye length, $\lambda_D \approx 0.1$ nm, which is two orders of magnitude shorter than observed. 1:2 ChCl:EG is a concentrated electrolyte (≈ 4.3 M, where M is defined as the moles of solute per volume of solution) and therefore this anomalously long decay length can be linked to underscreening-like behaviour reported by Smith *et al.*[14]

1:3 choline chloride:ethylene glycol

The normal force profile across a fresh sample of 1:3 ChCl:EG is shown in Figure S3(a). The oscillatory force at $D < 3$ nm is indicative of molecular layering between the surfaces. Two layers are observed with a thickness of 0.50 $\pm$ 0.01 nm. Similar to dry 1:2 ChCl:EG, the layer dimension is determined by the short axis of the choline cation, corresponding to a checkboard-like configuration of cations, anions and EG (see schematic in Figure S3(ci)).

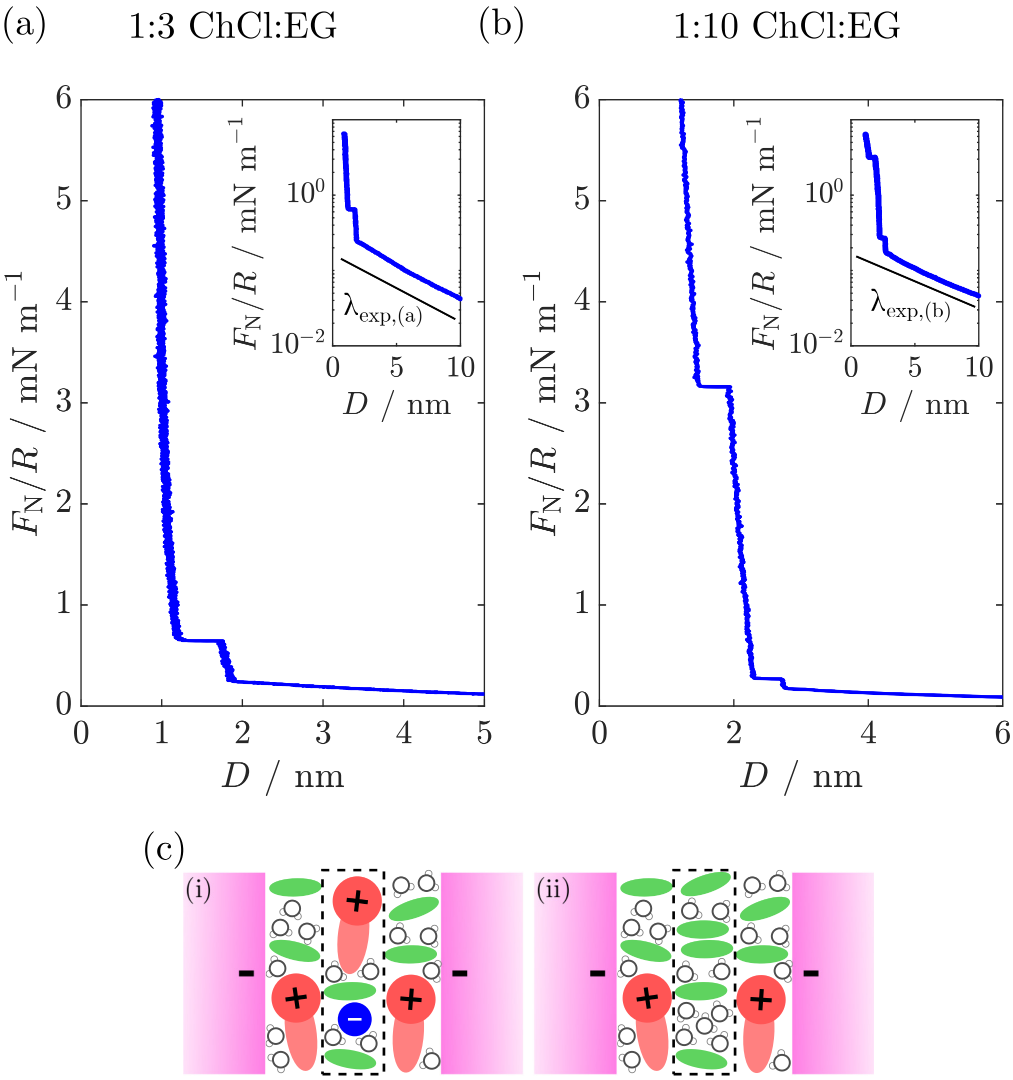

Figure S3 Representative normal force, F_N, normalised by the radius of curvature, R, as a function of surface separation, D, profile in fresh (a) 1:3 ChCl:EG and (b) 1:10 ChCl:EG. (a) The inset shows a log-linear representation of the normal force on approach. The solid black line indicates the gradient of the long-range exponential decay where $\lambda_{exp,(a)} = 5.8 \pm 0.4$ nm. (b) The inset shows a log-linear representation of the normal force on approach. The solid black line indicates the gradient of the long-range exponential decay where $\lambda_{exp,(b)} = 8.0 \pm 0.3$ nm. (c) Two proposed confined structure schematics (i) and (ii), determined using dimensional arguments. The negatively charged mica surfaces are shown in pink, ethylene glycol molecules in green, choline cations in red, chloride anions in blue, and water molecules in white and grey. The dashed black box indicates the squeeze-out repeat unit giving rise to steps in the force-distance profile.

1:10 choline chloride:ethylene glycol

Figure S3(b) shows the normal force profile across fresh 1:10 ChCl:EG. Molecular layering is observed with layer thicknesses of 0.425 $\pm$ 0.005 nm. The layer dimension is comparable to both the short axis of

the choline cation and the long axis of an ethylene glycol molecule. We propose two potential structures in Figure S3(c): (i) a mixed composition checkerboard-like layer and (ii) a pure EG layer. With a much higher concentration of EG in 1:10 ChCl:EG, we cannot discount the latter structure.

References

1. H. K. Christenson and R. G. Horn, *J. Colloid Interface Sci.*, 1985, **103**, 50–55.
2. Z. Chen, B. McLean, M. Ludwig, R. Stefanovic, G. G. Warr, G. B. Webber, A. J. Page and R. Atkin, *J. Phys. Chem. C*, 2016, **120**, 2225–2233.
3. R. M. Pashley, *J. Colloid Interface Sci.*, 1981, **80**, 153–162.
4. D. R. Lide, *CRC Handbook of Chemistry and Physics*, CRC Press, Boca Raton, FL, 84th edn., 2003.
5. R. Lhermerout, *Lubricants*, 2021, **9**, 69.
6. A. H. Turner and J. D. Holbrey, *Phys. Chem. Chem. Phys.*, 2019, **21**, 21782–21789.
7. J. E. Hallett, H. J. Hayler and S. Perkin, *Phys. Chem. Chem. Phys.*, 2020, **22**, 20253–20264.
8. S. Perkin, *Phys. Chem. Chem. Phys.*, 2012, **14**, 5052–5062.
9. M. Mezger, H. Schröder, H. Reichert, S. Schramm, J. S. Okasinski, S. Schöder, V. Honkimäki, M. Deutsch, B. M. Ocko, J. Ralston, M. Rohwerder, M. Stratmann and H. Dosch, *Science*, 2008, **322**, 424–428.
10. T. Cremer, M. Stark, A. Deyko, H.-P. Steinrück and F. Maier, *Langmuir*, 2011, **27**, 3662–3671.
11. R. M. Espinosa-Marzal, A. Arcifa, A. Rossi and N. D. Spencer, *J. Phys. Chem. Lett.*, 2014, **5**, 179–184.
12. M. H. Mamme, S. L. Moors, H. Terryn, J. Deconinck, J. Ustarroz and F. De Proft, *J. Phys. Chem. Lett.*, 2018, **9**, 6296–6304.
13. A. Pandey, R. Rai, M. Pal and S. Pandey, *Phys. Chem. Chem. Phys.*, 2014, **16**, 1559–1568.
14. A. M. Smith, A. A. Lee and S. Perkin, *J. Phys. Chem. Lett.*, 2016, **7**, 2157–2163.
